# Identification of a Novel Score for Adherence to the Mediterranean Diet That Is Inversely Associated with Visceral Adiposity and Cardiovascular Risk: The Chrono Med Diet Score (CMDS)

**DOI:** 10.3390/nu15081910

**Published:** 2023-04-15

**Authors:** Carlo De Matteis, Lucilla Crudele, Stefano Battaglia, Tiziana Loconte, Arianna Rotondo, Roberta Ferrulli, Raffaella Maria Gadaleta, Giuseppina Piazzolla, Patrizia Suppressa, Carlo Sabbà, Marica Cariello, Antonio Moschetta

**Affiliations:** Department of Interdisciplinary Medicine, University of Bari “Aldo Moro”, 70124 Bari, Italy; carlo.dematteis@uniba.it (C.D.M.); lucilla.crudele@uniba.it (L.C.); battagliastefano87@gmail.com (S.B.); locontenutrizionista@gmail.com (T.L.); ariannarotondo90@gmail.com (A.R.); ferrulliroberta@libero.it (R.F.); raffaella.gadaleta@uniba.it (R.M.G.); giuseppina.piazzolla@uniba.it (G.P.); patrizia.suppressa@uniba.it (P.S.); carlo.sabba@uniba.it (C.S.)

**Keywords:** nutrition, Mediterranean diet, biomarkers, score, cardiovascular risk, visceral adiposity

## Abstract

Adherence to the Mediterranean diet (MedDiet) leads to reduction of mortality from all causes, especially in subjects with cardiovascular disease, obesity, and diabetes. Numerous scores have been proposed to evaluate the adherence to MedDiet, mainly focused on eating habits. In this study, we verified whether existing validated MedDiet scores, namely, MEDI-LITE and the Mediterranean Diet Score (MDS), could be associated with visceral adiposity. Failing to find a significant association with adiposity, we proposed the validation of a new, easy-to-use adherence questionnaire, the Chrono Med-Diet score (CMDS). CMDS contains eleven food categories, including chronobiology of dietary habits and physical activity. Compared to the MEDI-LITE score and MDS, low values of CMDS are linked to increased waist circumference (WC) and dysmetabolic conditions. CMDS was also inversely correlated with cardiovascular risk (CVR), as well as Fatty Liver Index (FLI). In conclusion, the CMDS is a novel questionnaire to study the adherence to the MedDiet that, focusing on type and timing of carbohydrates intake, has the peculiar capability of capturing subjects with abdominal obesity, thus being an easy-to-use instrument of personalized medicine.

## 1. Introduction

### 1.1. Overview of the Mediterranean Diet

The Mediterranean diet (MedDiet) is an eating pattern scientifically described during the 20th century, coming from a millenary tradition among populations living by the Mediterranean Sea [1]. The original MedDiet was highly characterized by significant consumption of vegetables and fruit associated with a balanced weekly intake of whole grains, legumes, nuts, seeds, aromatic herbs, and extra-virgin olive oil (EVOO) as the most significant source of fat. EVOO has emerged as the leading fat product due to its significant content of monounsaturated fat (oleic acid and monounsaturated fatty acids—MUFA), which is considered responsible for its antiatherogenic characteristics [2,3], whilst lower-quality oils lack these antioxidant and anti-inflammatory capabilities. Furthermore, it is known for having a small consumption of meat (favoring lean meats, such as chicken, turkey, or rabbit) and its products, especially processed meats, as well as little-to-no consumption of butter or whole-fat dairy products. Still, the MedDiet is more than a simple dietary pattern, as it proposes regular physical activity and hydration as central for a healthy condition. Thence, these two items were originally put at the base of the overall scheme.

### 1.2. Modern Eating Patterns and Dysmetabolic Conditions

Currently, far from the original MedDiet, eating patterns have changed. Typical meals (breakfast, lunch and dinner) are difficult to clearly identify during the day because skipping meals and snacking have become more frequent, even in subjects with medical-assisted eating patterns and dietings. These eating styles can have various effects on cardiometabolic health, particularly obesity, lipid profile, insulin resistance, and blood pressure. Eating the greatest amount of the total calorie intake earlier in the day, whilst promoting consistent overnight resting periods and lower calories intake is recommended, as it possesses positive effects on risk factors for heart disease and type-2 diabetes [4]. The role of MedDiet in preventing many pathologic conditions has been highlighted in the current literature [5,6]. Originally, it was well known for improving overall body weight and Body Mass Index (BMI), while recently it has been significantly linked to beneficial effects on subjects affected by metabolic syndrome (MetS), as well as on its single determinants (increased waist circumference, blood pressure, triglycerides, glycaemia, or decreased HDL cholesterol), thus being crucial to confer a benefit against a wide range of pathologic conditions (i.e., type 2 diabetes, myocardial infarction, and other cardiovascular conditions, etc.) [7,8,9]. It has also been identified as a reliable alternative to low-protein diet in chronic kidney disease (CDK) patients (stage II and III according to KDIGO guidelines [10]). Furthermore, given its protective effects by reducing oxidative stress and inflammatory processes and avoiding angiogenesis and inflammation, the MedDiet is considered a significant and manageable method to fight cancer occurrence [11].

### 1.3. Adherence Scores for the Mediterranean Diet

As previously proven by the European Perspective Investigation into Cancer and Nutrition (EPIC) study, the MedDiet is, to date, the best model to prevent cancer, especially due to its flavonoids’ quota [12,13]. Emerging evidence has also underlined the strong correlation between MedDiet and gut microbiota composition: the MedDiet promotes eubiosis and microbial diversity, facilitating gut homeostasis and interfering with gut dysbiosis, leakiness, and the establishment of specific niches for opportunistic pathobionts [14]. In this scenario, many efforts have been made on the evaluation of MedDiet adherence via an easy-to-use score. There are several ways to identify adherence to the MedDiet, starting from the original pyramid, then ranging from general descriptions, a priori scoring systems, a posteriori dietary patterns, or characterizations based on food and nutrient content [15]. Over the last few years, a priori scores have emerged as the most popular ones, being the easiest to correlate with primary dietary outcomes. The adherence score is consequently calculated as the sum of points awarded for better intakes of wellbeing products, while negative or no points are allocated for health-harming food or habits. This scoring system, although easy to compute, tends to be dependent on population characteristics and can be compromised amid study-to-study comparisons. Among the proposed scores, the MedDiet Score (MDS), developed by Panagiotakos et al. in 2007, has been considered one of the most significant scores for its ease of application, [16]. In 2017, Sofi et al. produced a new score, the MEDI-LITE, using the MDS as reference in an Italian cohort. [17]. Both MDS and MEDI-LITE were very relatable to the MedDiet pyramid and for the prediction of the adherence to the Mediterranean diet pattern.

In the present study, we developed a new score, the Chrono Med-Diet score (CMDS), which significantly represents the adherence to MedDiet, as well as identifies increased risk for metabolic diseases. Furthermore, we studied the efficacy, accuracy, and specificity of CMDS throughout a series of comparisons with validated scores, namely, the MDS and the MEDI-LITE.

## 2. Materials and Methods

### 2.1. Study Participants

Patients’ recruitments, clinical, anthropometric, and biochemical measurements were recorded in the electronic health registry for Metabolic Diseases of Department of Interdisciplinary Medicine—Internal Medicine Division of University of Bari “Aldo Moro” (Bari, Italy) from January 2020 to November 2021. A total of 300 patients were enrolled in this study (150 females, 150 males). All study participants underwent a physical exam, a biochemical evaluation, and were asked to answer specific questions about adherence to MedDiet and lifestyle through three different questionnaires, namely, MDS, MEDI-LITE, and CMDS. MDS and MEDI-LITE were used as references. All questionnaires were administered in a row with standard operating procedures by trained personnel.

### 2.2. Clinical Assessment

Anthropometric assessment was performed using standardized procedures. Waist circumference (WC) was measured at the midpoint between the inferior part of the 12th costa and the anterior-superior iliac crests and was considered pathological according to the MetS 2006 International Diabetes Federation (IDF) definition if >94 cm in males and >80 cm in females [18]. BMI was computed as weight (kg) divided by the square of height (m) and BMI values (kg/m^2^) 25.0–29.9 and over 30.0 were considered as overweight and obesity conditions, respectively. Average systolic and diastolic blood pressures (BP) were derived for each patient from three different measurements using manual sphygmomanometer. Hypertension was identified as systolic arterial blood pressure (SAP) ≥ 140 mmHg, diastolic arterial blood pressure (DAP) ≥ 90 mmHg and/or treatment with antihypertensive agents. Liver function markers, such as AST (SGOT) and ALT (SGPT), were analyzed using cut-off limits of 37 U/L and 78 U/L, individually. The cardiovascular risk was assessed using the official Framingham Heart Study estimator for cardiovascular disease in the upcoming 10-years, adjusted for lipids [19]. Prediabetes (preDM) and Diabetes Mellitus (DM) were identified according to international criteria. PreDM was determined using the following criteria: HbA1c (percentage of glycosylated hemoglobin) ≥5.7% and ≤6.4% and fasting plasma glucose (FPG) ≥100 and ≤125 mg/dL. For DM, the criteria were: HbA1c (percentage of glycosylated hemoglobin) ≥ 6.5%, fasting plasma glucose (FPG) ≥ 126 mg/dL, and/or treatment for diabetes. To characterize dyslipidemia, the HDL-c cut-off was <40 mg/dL in men and <50 mg/dL in women. Furthermore, a triglycerides (TG) value of 150 mg/dL for both genders was considered pathological, whereas a total cholesterol level of ≥200 mg/dl was used for the diagnosis of hypercholesterolemia. We then commutated non-invasive indexes, which are reliable indicators of liver fibrosis [20]. Fatty Liver Index (FLI) was calculated as (e 0.953 × loge (triglycerides) + 0.139 × BMI + 0.718 × loge (GGT) + 0.053 × WC − 15.745)/(1 + e 0.953 × loge (TG) + 0.139 × BMI + 0.718 × loge (GGT) + 0.053 × WC − 15.745) × 100. The adopted cut-offs whereas follows: FLI < 30 to rule out liver steatosis and FLI > 60 to diagnose it [21]. Visceral Adiposity Index (VAI) was calculated as (WC/39.68 + (1.88×BMI)) × (TG/1.03) × (1.31/HDL) for men and as (WC/36.58 + (1.89 × BMI)) × (TG/0.81) × (1.52/HDL) for female patients, assuming VAI = 1 in healthy nonobese subjects with normal adipose distribution and normal TG and HDL levels [22]. Hepatic Steatosis Index was measured as 8 × ALT/AST ratio + BMI (+2, if DM; +2, if female). The cut-offs were as follows: HSI < 30 to rule out liver steatosis, HSI 30–36 to predict low to mild liver steatosis, and HSI > 36 to predict mild to severe liver steatosis [23]. Non-Alcoholic Fatty Liver Disease Fibrosis Score (NFS) was assessed as −1.675 + 0.037 × age + 0.094 × BMI + 1.13 × preDM or DM (yes = 1, no = 0) + 0.99 × AST/ALT ratio − 0.013 × platelet count − 0.66 × albumin. Patients with a score lower than −1.5 were classified as “low probability of advanced liver fibrosis”, and those with a score of at least −1.5 were classified as “intermediate or high probability of advanced liver fibrosis” [24].

### 2.3. Med Diet Score

The MDS was developed to characterize the adherence to the MedDiet pattern by assigning a score ranging from 0 to 5 to distinguish no to significant consumption of products included in the rationale of MedDiet, whereas for foods presumed to be far from the rationale, the opposite scores were assigned [16]. A specific category was dedicated to potato consumption, with a score of 5 points given for the recommended consumption of three to four portions per week, a score of 4 points given for one to two consumptions per week, and lower scores for other frequency of consumptions, weekly or daily calculated. Additionally, alcohol intake was considered, using wine glasses as reference. Maximum score (5 points) was given for <3 wine glasses drank daily; no points were assigned when drinking >7 wine glasses and scores ranging 1–4 were designated for consumption of three to seven wine glasses per day. The overall score ranged from 0 to 55 points, which indicated the maximum Mediterranean dietary pattern adherence (Figure S1).

### 2.4. MEDI-LITE Score

The MEDI-LITE has been introduced to evaluate adherence to the Mediterranean diet [17]. Nine food categories were examined: (1) fruit; (2) vegetables; (3) cereal grains; (4) legumes; (5) fish and fish products; (6) meat and meat products; (7) dairy products; (8) alcohol intake and (9) olive oil. Typical products included in the MedDiet were given a score of 2 points to the highest category of consumption, whereas 1 was given for the halfway category and none were assigned for the lowest. On the other hand, products not included in the Mediterranean diet (meat and meat products, dairy), a score of 2 was assigned to the lowest intake category, a score of 1 to the halfway category, and none were assigned to the highest category of consumption. Points were also given to alcohol and olive oil consumption. The overall score was achieved by the grand total of each category, ranging from 0 (low to no adherence) to 18 (high adherence) (Figure S2).

### 2.5. Biochemical Measurements

To assess biochemical markers for glucose and lipid metabolism, serum was collected after overnight fasting. Liver and renal function markers were also measured.

### 2.6. Statistical Analysis

Descriptive statistical analyses of study sample were performed, and results were expressed as mean ± standard error of the mean (SEM) and frequencies (%), based on the considered variable. Specifically, comparisons of socio-demographic and clinical variables between two groups were conducted with the t-test (for continuous variables) and the Pearson χ2 test (for categorical variables). The correlation between continuous variables was also analyzed using Spearman’s Correlation Coefficient (r). *p*-values lower than 0.05 were considered significant. The receiver operating characteristic (ROC) curve was performed to identify the capability of each parameter of detecting cardiovascular event conditions. Youden’s Index, or equivalently, the highest sensitivity + specificity, was used to determine the optimal cut-off of each variable for prediction of each condition. MEDI-LITE and MDS were used as reference scores. The sensitivity of CMDS was based on the ratio of true positives to true positives plus false negatives [25]. The cut-off point of 30 selected to differentiate Mediterranean diet adherents and non-adherents, which was the upper tertile of distribution, according to the criterion previously used by other studies for this purpose [17]. All statistical analyses were performed using the NCSS 12 Statistical Software, version 12.0.2018 (NCSS, LLC Company, Kaysville, UT, USA) and GraphPad Prism, version 9.4.0.673 (GraphPad Software; San Diego, CA, USA).

## 3. Results

### 3.1. Chrono Med-Diet Score

The Chrono Med-Diet Score has been developed after careful consideration of the MedDiet rationale [26,27], together with additions from the literature and recent guidelines [2,28]. Eleven food categories were considered to determine the overall score, based on a daily to weekly intake: (1) fruit, (2) vegetables, (3) legumes, (4) farinaceous products (i.e., bread, pasta, cookies), (5) grain cereals, (6) fish, (7) meat and meat products, (8) milk and dairy products, (9) olive oil, (10) butter, margarine and lard, and (11) alcohol intake.

Along with these categories, two more were added to further characterize the eating habits and overall health status of the study participants: the time of farinaceous products intake and the physical activity. A score of 2 points was given to the highest consumption of canonical Mediterranean diet products (i.e., fruit, vegetables, legumes, fish, and olive oil), whereas a score of 1 point was assigned to the halfway category and a score of 0 points was given to the lowest intake for such products. On the other hand, meat, meat products, milk, and milk-derived products were assigned 2 points when eaten less than 1 time per day to 0 points when eaten more than 1.5 times per day.

Farinaceous products were given 2 points for consumption of one portion per day, whereas −1 point was assigned when eating more than 1.5 portions per day. Time of farinaceous consumption was also considered a standalone category. An intake by 3pm was given 1 point, while by 7pm it was assigned −1 point and −2 points if the farinaceous was eaten in the evening. The combined consumption in both lunch and dinner in the same day was given −4 points. Cereals were assigned 1 point per maximum intake and 0 points for consumption of less than 1 portion per day.

**Figure 1:**
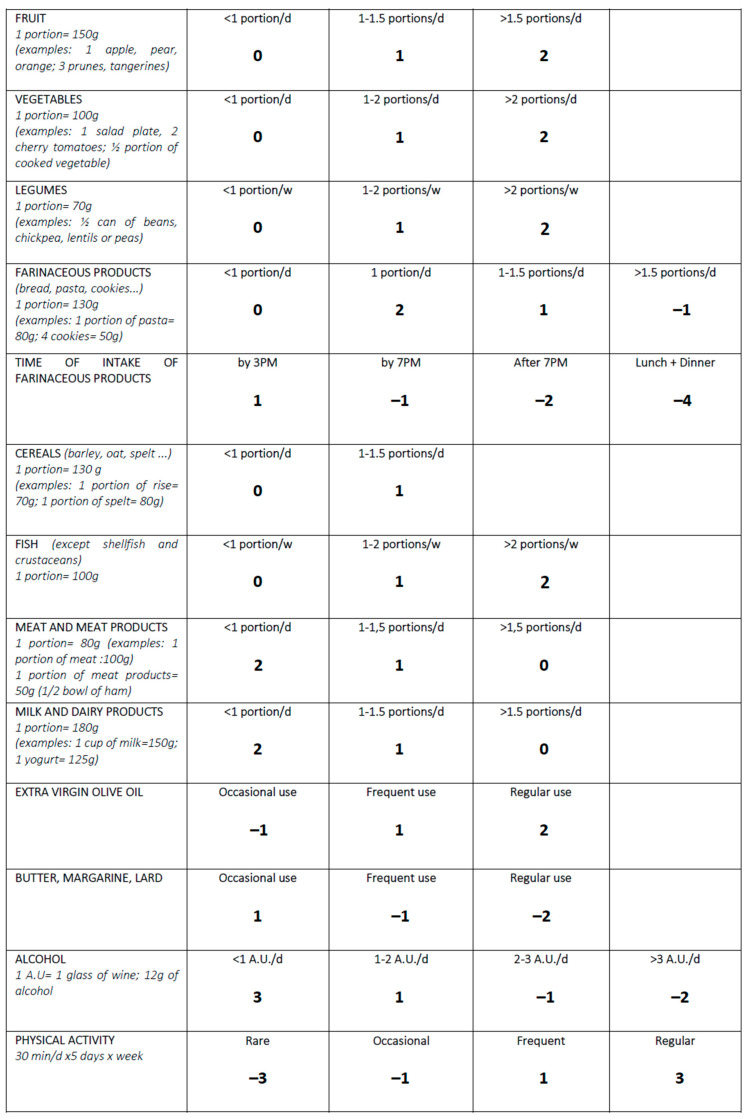
*The Chrono Med Diet Score.* Abbreviations: day, d; week, w; post meridian, PM.

The use of fats, such as butter, margarine, and lard, were assessed by assigning 1 point in case of occasional use, −1 point for frequent use, and −2 points for referred regular use. Alcohol intake was carefully considered using alcohol units (1 alcohol unit = 12 g of alcohol). A score of three points was assigned to a consumption of <1 U.A. per day, ranging to −2 points assigned to >3 U.A. per day. Physical activity was considered following the WHO guidelines [29], ranging from a score of 3 points for regular physical activity to −3 points in case of rare to none referred activity. The overall score ranged from −13 to 25 points, which indicated the best adherence to this new mixed rationale (Figure 1).

**Figure 2:**
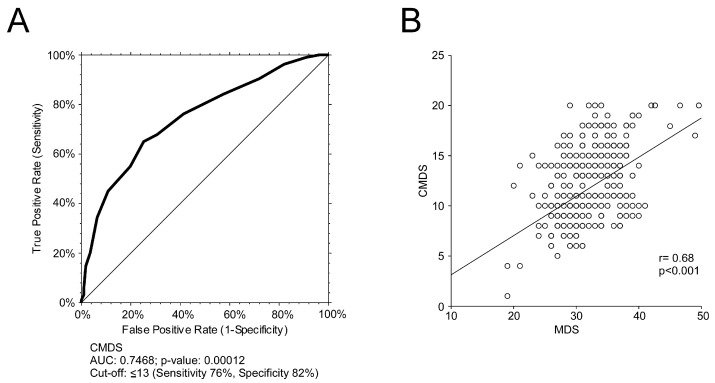
Discriminative power of the CMDS to identify adherence to the MedDiet using the MDS as reference score. The correlation analysis was estimated using Spearman Correlation Coefficient (r); (*p*) indicates statistical significance. Each dot represents a single subject. (**A**) ROC curve of CMDS and MDS to identify adherence to the MedDiet using MDS as reference. The cut-off point for the reference score was 30, in accordance with previous studies, which consider that the upper tertile represents greater adherence to the MedDiet; (**B**) Correlation analysis of CMDS and MDS overall score.

To validate the discriminative capability of CMDS in identifying subjects adherent to the MedDiet (Figure 2), we performed a ROC curve, considering the MDS as reference method. CMDS showed a significant discriminative power (AUC 0.7468, 95% CI 0.6122–1.1835) (Figure 2A). The best CMDS value to discriminate adherent and non-adherent individuals was 13 (76% sensitivity, 82% specificity). Nonetheless, to further evaluate the predictive capabilities of the proposed score, we performed a correlation analysis between CMDS and MDS (Figure 2B).

The questionnaire is also available at www.chronomeddiet.org (accessed on 12 March 2023) for free consultation and personal assortment.

### 3.2. Clinical Characterization of the Study Population

Baseline characteristics of the study population are shown in Table 1.

We enrolled 300 patients (150 females and 150 males). The mean baseline BMI and WC suggested that patients were mostly overweight with a mean WC found positive for MetS diagnosis, according to IDF criterion. However, other metabolic biomarkers, such as glucose, HbA1c, total cholesterol, and HDL-c, were within the physiological range. Liver serum markers (GGT, AST, ALT) and non-invasive scores of fibrosis were also not significantly altered among the overall population. As for MedDiet adherence scores, the MDS and MEDI-LITE both were suggestive of moderate adherence to the principles of MedDiet.

### 3.3. Association between MDS and MEDI-LITE Cut-Offs’ Identification and Increased WC

**Figure 3:**
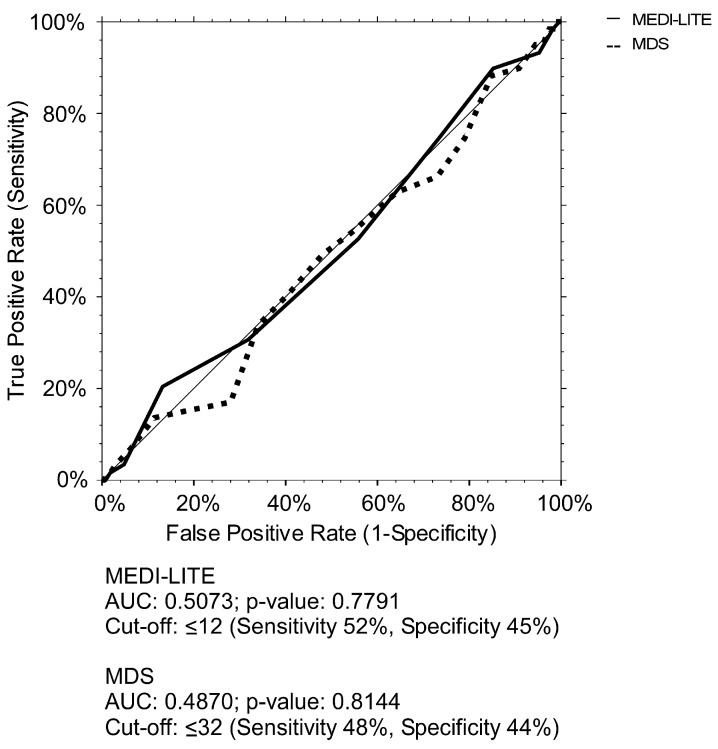
ROC curves of MDS and MEDI-LITE for increased waist circumference. ROC curves of MEDI-LITE score and MDS for increased waist circumference according to MetS IDF definition (≥94 cm in men and ≥80 in women).

To further study our baseline findings of increased WC and the capability of MDS and MEDI-LITE to discriminate visceral obese individuals, we plotted a ROC curve for both scores (Figure 3). Our findings highlight no significance for MEDI-LITE (AUC = 0.5073, 95% CI 0.4197–0.5855 and MDS (AUC = 0.4870, 95% CI 0.3961–0.5619). Indeed, also the best values to discriminate visceral obese patients lack sensibility and specificity.

Nonetheless, to evaluate the probability of proposed adherence cut-offs to identify metabolic morbidity, we divided our study population according to them, considering subjects with proposed high adherence as controls. Neither group presented significant differences compared to them. Specifically, in the MDS group we found higher LDL-c in subjects with MDS ≤ 32, as well as increased GGT. Liver steatosis scores, such as Fatty Liver Index (FLI), Visceral Adiposity Index (VAI), and NAFLD score (NFS), were also found to be significantly higher. On the other hand, in the MEDI-LITE group we did not find any significantly different condition except for GGT, which was found higher in patients with MEDI-LITE ≤ 12, demonstrating that cut off values are not able to discriminate pathological WC (Table 2 and Table 3).

### 3.4. Associaton between CMDS, Increased WC and Dysmetabolic Conditions

**Figure 4:**
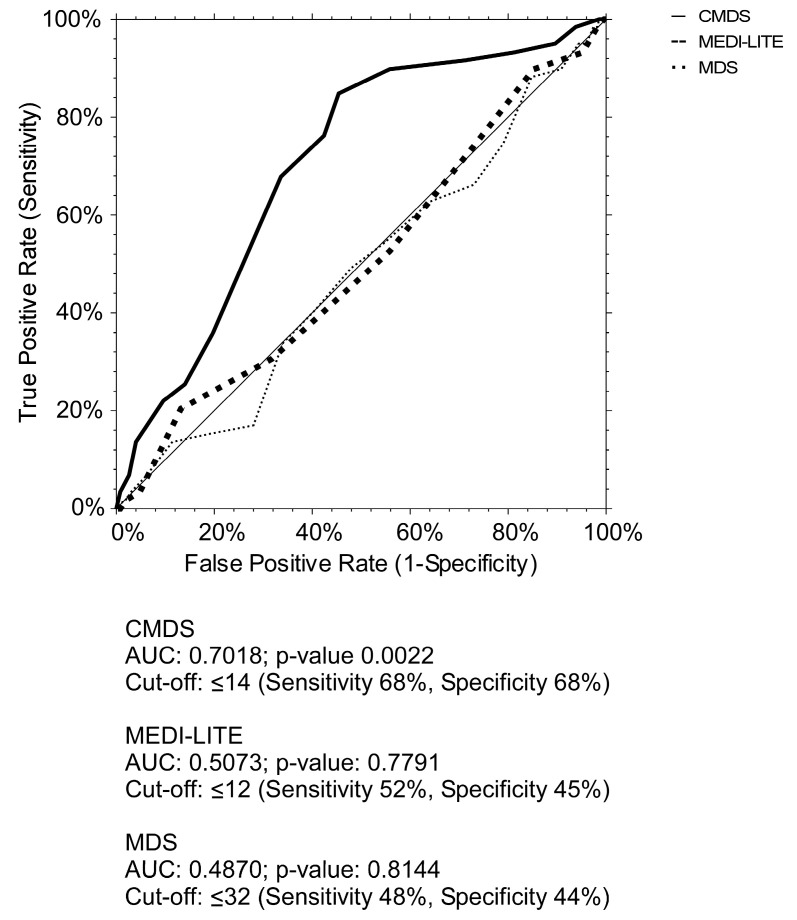
ROC curve of CMDS, MEDI-LITE, and MDS for increased waist circumference. ROC curves characteristics of CMDS, MEDI-LITE score, and MDS for increased WC according to MetS IDF definition (≥94 cm in men and ≥80 in women).

To evaluate the association between CMDS, increased WC and dysmetabolic conditions, we performed CMDS ROC curve comparing it to MDS and MEDI-LITE ones. Our findings show that CMDS had a more significant power to discriminate increased WC (AUC = 0.7018, 95% CI 0.6437–0.7995, *p*-value < 0.005). The optimal cut-off value to identify increased WC was 14, with a sensitivity of 68% and a specificity of 68% (Figure 4).

Moreover, we broke down our study population using the CMDS cut-off value given by the ROC curve. Our data show that subjects with CMDS ≤ 14 had pathologically higher metabolic markers, such as BMI, total cholesterol, TG, glucose, HbA1c, LDL-c, as well as decreased HDL-c and 25-OH Vitamin D, compared to subjects with CMDS > 14. Liver markers, such as GGT and ALP, were also significantly altered in patients with CMDS ≤ 14, along with considerably higher liver steatosis scores, such as FLI, VAI, HSI, and NFS. These results highlight the ability of CMDS and its association with increased WC and dysmetabolic conditions (Table 4).

### 3.5. Correlation between MedDiet Adherence Scores, Clinical and Biochemical Parameters for Metabolic Disorders

To better characterize differences between CMDS, MDS, and MEDI-LITE and understand the interaction of these scores with clinical and biochemical parameters, we calculated the Spearman correlation. Results show that CMDS significantly correlates with all major metabolic indicators, whereas MDS and MEDI-LITE lack any significance, except in the correlation with 25-OH-Vitamin D.

Interestingly, CMDS especially negatively correlates with WC, TG, and glucose, along with FLI, VAI, HSI, and NFS (*p* < 0.001) whereas MDS and MEDI-LITE lacked a significant correlation with all the markers. HbA1c also negatively correlates with CMDS (*p* < 0.005), while it is not associated with MDS and MEDI-LITE (*p* = 0.2 and *p* = 0.06 respectively).

CMDS also showed a significant correlation with CVR and BMI, proving that the adherence to MedDiet has an impact on biometric data and cardiovascular estimates. All results of the correlation analysis are shown in Table 5.

**Figure 5:**
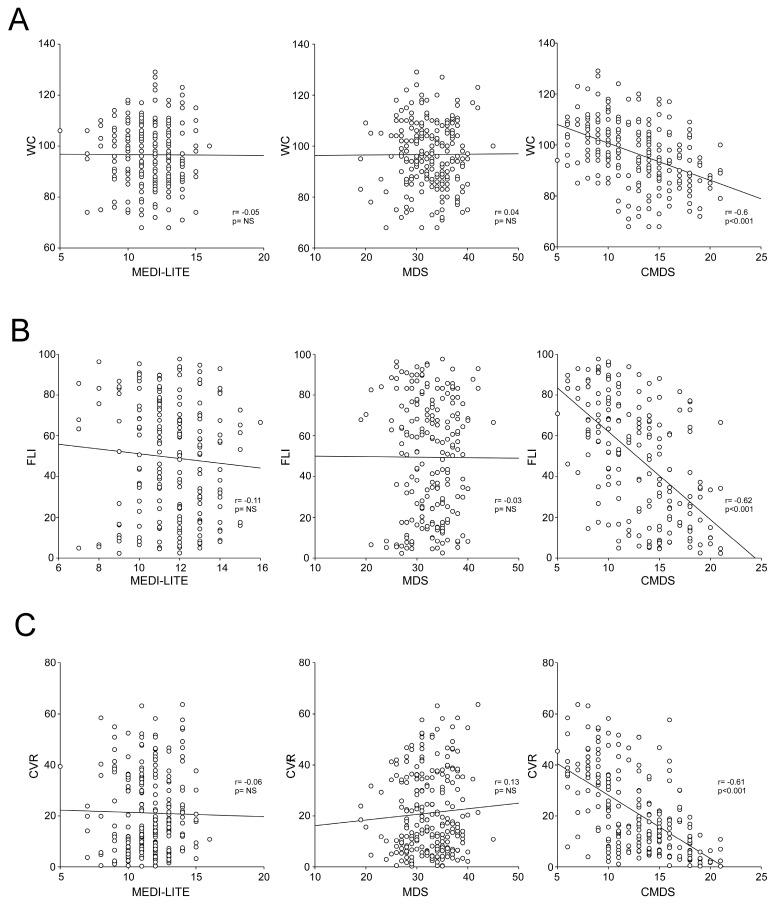
Correlation between Chrono Med Diet Score, MEDILITE score, and Med Diet Score with visceral adiposity, liver steatosis, and cardiovascular risk. The correlation analysis was estimated using Spearman Correlation Coefficient (r); (*p*) indicates statistical significance. Each dot represents a single subject. (**A**) Correlation analysis of waist circumference (WC) with MEDI-LITE, MDS, and CMDS. (**B**) Correlation analysis of Fatty Liver Index (FLI) with MEDI-LITE, MDS, and CMDS. (**C**) Correlation analysis of cardiovascular risk score (CVR) with MEDI-LITE, MDS, and CMDS.

To corroborate the impact of CMDS among these scores and its association with visceral adiposity, liver steatosis and cardiovascular risk, we performed a graphical demonstration of the correlations between the three adherence scores and WC (Figure 5A), FLI (Figure 5B) and CVR (Figure 5C).

### 3.6. Data Distribution According to Scores’ ROC-Identified Cut-Offs for Major Dysmetabolic Biomarkers

**Figure 6:**
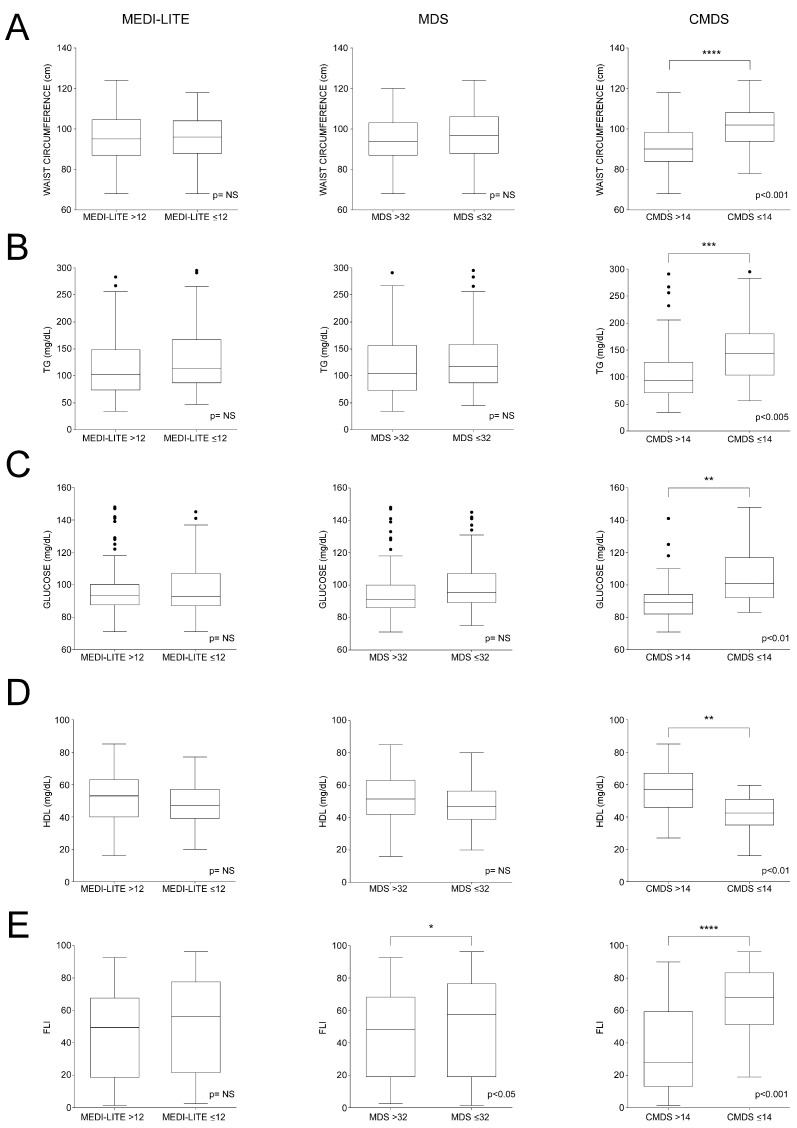
Dysmetabolic-related biomarkers in subgroups population identified according to MEDI-LITE, MDS, and CMDS cut-offs. Box plots show median (second quartile), first and third quartile. Tukey whiskers reach 1.5 times the interquartile distance or the highest or lowest point, whichever is shorter. Any data beyond whiskers are shown black dots. Comparisons were performed using Students’ *t* test. * *p* < 0.05, ** *p* < 0.01, *** *p* < 0.005; **** *p* < 0.001 indicates statistical significance. Abbreviations: triglycerides, TG; high-density lipoprotein cholesterol, HDL; Fatty Liver Index, FLI. (**A**) Box plots of waist circumference according to MEDI-LITE, MDS and CMDS cut-offs; (**B**) Box plots of triglycerides according to MEDI-LITE, MDS and CMDS cut-offs; (**C**) Box plots of glucose according to MEDI-LITE, MDS and CMDS cut-offs; (**D**) Box plots of high-density lipoprotein cholesterol according to MEDI-LITE, MDS and CMDS cut-offs; (**E**) Box plots of Fatty Liver Index according to MEDI-LITE, MDS and CMDS cut-offs.

We studied WC, TG, glucose, HDL, and FLI in the subpopulations identified according to their MEDI-LITE, MDS, and CMDS values (Figure 6). Interestingly, we observed that, between subgroups identified according to MEDI-LITE ≤ 12 and MDS ≤ 32, a significant difference was found only for HDL-c levels (*p* < 0.05), (Figure 6D), this difference being more significant in CMDS subpopulations.

In all other analysis, WC (Figure 6A), triglycerides (Figure 6B), glucose (Figure 6C), and FLI (Figure 6E) did not significantly differ in subgroups identified through MEDI-LITE and MDS cut-offs, whereas CMDS ≤ 14 highlighted a powerful association with dysmetabolism, especially to describe increased visceral adiposity and liver steatosis, thus confirming a strong correlation between CMDS and clinical parameters of metabolic disorders.

## 4. Discussion

In this cross-sectional, gender-balanced cohort study, we propose new criteria to discriminate adherence to the MedDiet lifestyle. On the frame of well-established adherence scores, such as the MDS and MEDI-LITE, as well as the literature that followed [30], we standardized a simple, easy-to-use survey that was based on the literature [31] that evaluates the adherence not only to MedDiet principles, but also overall wellbeing and reduced visceral adiposity.

Indeed, MedDiet, differently from other dietary regimens, can be considered a lifestyle, rather than just a diet, and it is inscribed on the representative list of the Intangible Cultural Heritage of Humanity from the UNESCO [32].

Moreover, MedDiet has been established as a significant contributor to the reduced mortality from all causes, especially cardiovascular disease, obesity, MetS, diabetes, and other chronic cardiometabolic conditions [33,34,35]. However, it remains unclear if one or more single habits of MedDiet have the heaviest role in conferring protection from mortality and the above-mentioned diseases. To evaluate adherence to MedDiet, more than 70 original studies have proposed its interpretation in the last years [36], using different approaches, such as classical scoring systems, modern dietary patterns, and food combinations, but they failed to accurately predict the burden of diseases associated to unhealthy dietary regimens and lifestyle. For instance, one of the main limitations of these scores is the evaluation of MedDiet adherence based only on food consumption. Therefore, it is necessary to have an easy-to-use adherence questionnaire that contains information on food as well as lifestyle habits.

Starting from items of the two reference scores, we proposed a questionnaire with a peculiar approach to cereals (dividing products, such as barley and oat from bread and cookies) and fats (EVOO versus butter and margarine [37,38]), which represents a novelty in this kind of scores. Intriguingly, this new scoring system is associated with a variety of conditions, ranging from visceral obesity, identified by a simple marker such as WC, to dyslipidemia, glucose intolerance, increased cardiovascular disease risk, and liver steatosis, whereas MDS and MEDI-LITE did not highlight the same results and were not relatable with increased WC as well as other pathologic conditions in the study population.

Ross et al. recently argued that BMI alone is not sufficient to properly assess or manage the cardiometabolic risk associated with increased adiposity in adults and associated the decrease in WC to a critical treatment target for reducing overall adverse health conditions in both genders [39]. Our findings highlight healthier WC in patients with higher scores of CMDS (r = −0.6, *p* < 0.001), whereas MDS and MEDI-LITE do not clarify any change in measurements, even in high-adherence subjects, while also not suggesting a great sensitivity or specificity to be associated with increased WC. This difference allows the CMDS to highlight potentially critical conditions far better than BMI, which is found to be in the same range of overweight, even though found to be statistically different. These findings could be associated with the choice to negatively consider the intake of starchy products in the evening or if subjects indicated consumption of bread or cookies during lunch and dinner combined. Several studies demonstrated that timing of eating [4,40,41,42] can be synchronized with different organs (i.e., stomach, gut, liver, adipose tissue), as well as to food digestion, absorption, or metabolism. Moreover, in addition to excess caloric intake, timing of eating may be decisive in fat accumulation and mobilization and can affect the effectiveness of weight loss treatments. Consensus data indicate that eating more of the day’s total energy intake by midday is associated with a lower risk of increased visceral adiposity, whereas consuming more in the evening is associated with visceral obesity and its systemic implications [43,44]. In the present study, considering the implications of work habits, we identified 3 PM as the latest time of intake recognized as healthy, especially for starchy products.

Furthermore, we differentiated cereals intake in two different categories. As previously pointed out by Aune et al. [45] and Korem et al. [46], whole grain products represent approximately 56% of the daily energy intake, and, for this reason, they have been recently targeted as one of the most significant components of the global food patterns to be studied. Glycemic response to this consumption must be controlled, as it is person-specific, whereas bread types, especially, are not that different. In this scenario, our study provides a first step in recognizing that whole grain products should not be considered together while studying dietary patterns, as almost 80% of subjects in the present study that showed increased WC admitted an intake of bread both at lunch and dinner.

In relation to drinking, considering the variety of studies conducted on this matter [47,48], we characterized the alcohol intake negatively if >2 alcohol units (A.U.) per day, since there is still significant debate on the ideal definition of standard alcoholic drink [49]. Our approach was useful, since most subjects enrolled in the present study (56%) answered to use less than 2 A.U. per day.

Furthermore, the role of physical activity was also carefully considered to strengthen the overall quality of the score. Previous MedDiet scores did not contemplate physical activity, although it sits at the bottom of the original pyramid, and it is crucial for healthy aging and reducing cardiovascular disease risk [50].

## 5. Conclusions

Taken together, our findings show that CMDS is a reliable, easy-to-use adherence questionnaire that contains important information on food, as well as lifestyle habits, in a modern approach. Results of this study are the first to consider type of food and its portions along with time of intake, creating a rapid and comprehensive score that can be administered in every setting at virtually no cost. The finding that, although a correlation between CMDS and MDS exists, the CMDS alone is associated with WC and metabolic derangement biomarkers, strengthens our hypothesis that the type of carbohydrates along with the time of their intake and physical activity are major actors in dumping adipogenesis and visceral fat depots.

In conclusion, the CMDS can be considered an easy-to-use instrument of personalized medicine to characterize conditions and behaviors that are the main responsible of adiposity-associated metabolic derangement.

## Figures and Tables

**Table 1:** Clinical characterization of the study population.

Clinical Variable	N = 300 (150M:150F)
Age (Years)	59.6 ± 0.8
BMI (Kg/m^2^)	27.1 ± 5.6
Waist circumference (cm)	97.2 ± 0.8
SBP (mmHg)	128.9 ± 0.9
DBP (mmHG)	84.4 ± 1.9
Erythrocytes (10^6^/µL)	4.8 ± 0.1
WBC (10^3^/µL)	6.3 ± 0.1
Hemoglobin (g/dL)	13.8 ± 0.1
Neutrophils (%)	59.2 ± 0.5
Eosinophils (%)	3.1 ± 0.3
Basophils (%)	0.6 ± 0.1
Lymphocytes (%)	30.7 ± 0.5
Monocytes (%)	6.4 ± 0.2
Platelet count (10^^6^/μL)	231.3 ± 3.6
Total cholesterol (mg/dL)	178.8 ± 2.4
HDL-c (mg/dL)	55.2 ± 1.1
LDL-c (mg/dL)	102.1 ± 2
TG (mg/dL)	117.6 ± 3.7
Glucose (mg/dL)	98.7 ± 1.6
HbA1c (mmol/mol)	39.9 ± 1
25-OH Vitamin D(ng/mL)	22.6 ± 0.6
Hs-CRP (mg/L)	4.7 ± 0.5
GGT (U/L)	35.2 ± 1
AST (U/L)	20.9 ± 0.6
ALT (U/L)	28.3 ± 1
ALP (U/L)	63.9 ± 1.4
CVR (Framingham)	16.77 ± 1
FLI	50 ± 2
VAI	4.1 ± 0.2
HSI	33.6 ± 0.4
NFS	−1 ± 0.1
MED DIET SCORE	32.2 ± 0.3
MEDI-LITE SCORE	12.8 ± 0.2

Data are presented as mean ± SEM (standard error of the mean). Abbreviations: body mass index, BMI; systolic blood pressure, SBP; white blood cells, WBC; high-density lipoprotein cholesterol, HDL-c; low-density lipoprotein cholesterol, LDL-c; triglycerides, TG; glycosylated hemoglobin, HbA1c; high-sensitivity C reactive protein Hs-CRP; gamma-glutamyltransferase, GGT; aspartate transaminase, AST; alanine transaminase, ALT; alkaline phosphatase, ALP; cardiovascular risk, CVR; Fatty Liver Index, FLI; Visceral Adiposity Index, VAI; Hepatic Steatosis Index, HIS; NAFLD Score, NFS.

**Table 2:** Comparison of subgroups identified according to Med Diet Score (MDS) above and under the ROC-identified cut-off for pathologic visceral obesity assessed as increased waist circumference.

Clinical Variable	MDS > 32 N = 136 (61M:75F)	MDS ≤ 32 N = 164 (89M:75F)	*p*-Value
WC (cm)	90.7 ± 1.9	92.1 ± 2.8	ns
BMI (Kg/m^2^)	27.2 ± 0.4	28.8 ± 0.5	ns
SBP (mmHg)	127 ± 1.8	129.8 ± 2	ns
DBP (mmHg)	85.4 ± 0.8	84.1 ± 0.9	ns
Erythrocytes (10^6^/µL)	4.7 ± 0.1	4.8 ± 0.1	ns
WBC (10^3^/µL)	6 ± 0.2	6.1 ± 0.1	ns
Hemoglobin (g/dL)	13.8 ± 0.1	13.8 ± 0.1	ns
Neutrophils (%)	59.1 ± 0.8	59.6 ± 0.7	ns
Eosinophils (%)	2.9 ± 0.2	2.6 ± 0.1	ns
Basophils (%)	0.6 ± 0.1	0.6 ± 0.1	ns
Lymphocytes (%)	30.9 ± 0.7	31.2 ± 0.6	ns
Monocytes (%)	6.3 ± 0.2	6.1 ± 0.1	ns
Platelet count (10^6^/μL)	228.8 ± 5.2	233.5 ± 5	ns
Total cholesterol (mg/dL)	176.2 ± 3.3	181.9 ± 3.3	ns
HDL-c (mg/dL)	51.8 ± 1.3	53.2 ± 1.4	ns
LDL-c (mg/dL)	99 ± 2.9	104.8 ± 2.9	<0.05
TG (mg/dL)	116 ± 5.6	120.6 ± 5.2	ns
Glucose (mg/dL)	98.5 ± 2.1	102.4 ± 2.3	ns
HbA1c (mmol/mol)	40.2 ± 1.2	40.4 ± 1.3	ns
25-OH Vitamin D(ng/mL)	22.6 ± 0.8	22 ± 1	ns
Hs-CRP (mg/L)	4.1 ± 0.5	4.2 ± 0.5	ns
GGT (U/L)	33.7 ± 2.8	37.4 ± 3.1	<0.05
AST (U/L)	23.86 ± 0.8	24.1 ± 3.1	ns
ALT (U/L)	31.1 ± 1.6	30.19 ± 1.4	ns
ALP (U/L)	72.9 ± 2	75.63 ± 2.1	ns
CVR (Framingham)	17.2 ± 3.2	19.98 ± 1.2	ns
FLI	43 ± 7.3	55.66 ± 2.9	<0.05
VAI	3 ± 0.8	4.01 ± 0.4	<0.05
HSI	36.7 ± 0.8	37.39 ± 0.6	ns
NFS	−1 ± 0.2	−0.73 ± 0.7	<0.05

Data are presented as mean ± SEM (standard error of the mean). Abbreviations: Med diet score, MDS; Body Mass Index, BMI; systolic blood pressure, SBP; white blood cells, WBC; high-density lipoprotein cholesterol, HDL-c; low-density lipoprotein cholesterol, LDL-c; triglycerides, TG; glycosylated hemoglobin, HbA1c; high-sensitivity C reactive protein Hs-CRP; gamma-glutamyltransferase, GGT; aspartate transaminase, AST; alanine transaminase, ALT; alkaline phosphatase, ALP; Cardiovascular Risk, CVR; Fatty Liver Index, FLI; Visceral Adiposity Index, VAI; Hepatic Steatosis Index, HIS; NAFLD Score, NFS.

**Table 3:** Comparison of subgroups identified according to MEDI-LITE above and under the ROC-identified cut-off of 12 for visceral obesity assessed as increased waist circumference.

Clinical Variable	MEDI-LITE > 12 N = 104 (49M:55F)	MEDI-LITE ≤ 12 N = 196 (101M:95F)	*p*-Value
WC (cm)	89.3 ± 2.4	92.7 ± 1.7	ns
BMI (Kg/m^2^)	28.3 ± 0.6	27.9 ± 0.4	ns
SBP (mmHg)	128.3 ± 1.3	128.7 ± 1.8	ns
DBP (mmHg)	85 ± 1.1	86 ± 2.2	ns
Erythrocytes (10^6^/µL)	4.7 ± 0.1	4.8 ± 0.1	ns
WBC (10^3^/µL)	6.1 ± 0.1	6.1 ± 0.1	ns
Hemoglobin (g/dL)	14 ± 0.1	13.7 ± 0.1	ns
Neutrophils (%)	59.4 ± 1	59.4 ± 0.6	ns
Eosinophils (%)	2.8 ± 0.2	2.7 ± 0.1	ns
Basophils (%)	0.5 ± 0.1	0.6 ± 0.1	ns
Lymphocytes (%)	30.7 ± 0.8	31.2 ± 0.5	ns
Monocytes (%)	6.4 ± 0.2	6.2 ± 0.1	ns
Platelet count (10^6^/μL)	232.5 ± 6.3	231.1 ± 4.4	ns
Total cholesterol (mg/dL)	181.7 ± 4.1	178.1 ± 2.9	ns
HDL-c (mg/dL)	53.6 ± 1.9	54.4 ± 1.2	ns
LDL-c (mg/dL)	105 ± 3.6	100.8 ± 2.5	ns
TG (mg/dL)	118 ± 6.	118.6 ± 4.8	ns
Glucose (mg/dL)	103.7 ± 3.5	98.9 ± 1.6	ns
HbA1c (mmol/mol)	41.3 ± 1.7	39.9 ± 1	ns
25-OH Vitamin D(ng/mL)	24.1 ± 1.1	22.1 ± 0.8	ns
Hs-CRP (mg/L)	4.2 ± 0.4	4.1 ± 0.4	ns
GGT (U/L)	32.1 ± 3.4	37.1 ± 2.6	<0.05
AST (U/L)	22.9 ± 1	24.5 ± 0.8	ns
ALT (U/L)	28.7 ± 1.4	31.6 ± 1.4	ns
ALP (U/L)	72.5 ± 2.7	73.1 ± 1.7	ns
CVR (Framingham)	21.3 ± 1.7	20.2 ± 1.2	ns
FLI	51.9 ± 3.4	51.7 ± 3.7	ns
VAI	4.1 ± 0.3	4.2 ± 0.2	ns
HSI	36.9 ± 0.8	36.3 ± 0.5	ns
NFS	−0.9 ± 0.22	−1.1 ± 0.1	ns

Data are presented as mean ± SEM (standard error of the mean). Abbreviations: Body Mass Index, BMI; systolic blood pressure, SBP; white blood cells, WBC; high-density lipoprotein cholesterol, HDL-c; low-density lipoprotein cholesterol, LDL-c; triglycerides, TG; glycosylated hemoglobin, HbA1c; high-sensitivity C reactive protein Hs-CRP;; gamma-glutamyltransferase, GGT; aspartate transaminase, AST; alanine transaminase, ALT; alkaline phosphatase, ALP; cardiovascular risk, CVR; Fatty Liver Index, FLI; Visceral Adiposity Index, VAI; Hepatic Steatosis Index, HIS;NAFLD Score, NFS.

**Table 4:** Baseline characteristics comparison of patients divided in two groups according to Chrono-Med Diet cut-off to identify increased visceral adiposity.

Clinical Variable	CMDS >14 N = 97 (40M:57F)	CMDS ≤ 14 203 (110M:93F)	*p*-Value
WC (mmHg)	87.9 ± 2.1	97.4 ± 0.8	<0.01
BMI (Kg/m^2^)	25.1 ± 0.5	29.7 ± 5.5	<0.05
SBP (mmHg)	122 ± 1.4	131.6 ± 1	<0.05
DBP (mmHg)	81.3 ± 1.7	84.1 ± 1	ns
Erythrocytes (10^6^/µL)	4.8 ± 0.1	4.8 ± 0.1	ns
WBC (10^3^/µL)	5.8 ± 0.2	6.5 ± 0.1	ns
Hemoglobin (g/dL)	13.7 ± 0.1	13.9 ± 1	ns
Neutrophils (%)	59.2 ± 1	59.5 ± 0.6	ns
Eosinophils (%)	2.9 ± 0.2	2.6 ± 0.1	ns
Basophils (%)	0.6 ± 0.3	0.6 ± 0.3	ns
Lymphocytes (%)	31 ± 0.9	31.1 ± 0.5	ns
Monocytes (%)	6.3 ± 0.2	6.2 ± 0.1	ns
Platelet count (10^6^/μL)	227.3 ± 5.6	203.1 ± 4.6	<0.05
Total cholesterol (mg/dL)	179.6 ± 4.1	191.9 ± 3	<0.05
HDL-c (mg/dL)	59.1 ± 1.4	49.6 ± 1.2	<0.01
LDL-c (mg/dL)	104.1 ± 3.7	111 ± 2.5	ns
TG (mg/dL)	89.9 ± 4.6	131.6 ± 4.9	<0.005
Glucose (mg/dL)	92.3 ± 2.5	106.4 ± 1.9	<0.01
HbA1c (mmol/mol)	35.2 ± 1.3	43.5 ± 1.1	<0.05
25-OH Vitamin D(ng/mL)	26 ± 1.4	18.4 ± 0.7	<0.05
Hs-CRP (mg/L)	3.7 ± 0.3	5 ± 0.4	ns
GGT (U/L)	32.6 ± 4	39 ± 2.4	<0.05
AST (U/L)	22.7 ± 1	26.6 ± 0.8	ns
ALT (U/L)	29.4 ± 2	31.3 ± 1.3	ns
ALP (U/L)	70.5 ± 2.3	81.2 ± 1.8	<0.05
CVR (Framingham)	12.1 ± 1.2	24.4 ± 1.3	<0.01
FLI	33.9 ± 2.8	60.8 ± 2.2	<0.001
VAI	2.8 ± 0.2	4.8 ± 0.2	<0.05
HSI	33.6 ± 0.6	39.9 ± 0.5	<0.05
NFS	−1.9 ± 0.2	−0.5 ± 0.1	<0.005

Data are presented as mean ± SEM (standard error of the mean). Abbreviations: Chrono Med Diet Score, CMDS; Body Mass Index, BMI; systolic blood pressure, SBP; white blood cells, WBC; high-density lipoprotein cholesterol, HDL-C; low-density lipoprotein cholesterol, LDL-C; triglycerides, TG; glycosylated hemoglobin, HbA1c; high-sensitivity C reactive protein Hs-CRP; gamma-glutamyltransferase, GGT; aspartate transaminase, AST; alanine transaminase, ALT; alkaline phosphatase, ALP; cardiovascular risk, CVR; Fatty Liver Index, FLI; Visceral Adiposity Index, VAI; Hepatic Steatosis Index, HIS;NAFLD Score, NFS.

**Table 5:** Analysis of correlations between Mediterranean diet scores, clinical and biochemical parameters.

Clinical Variable	MEDI-LITE		MDS		CMDS	
	Spearman (r)	*p*-Value	Spearman (r)	*p*-Value	Spearman (r)	*p*-Value
BMI (Kg/m^2^)	−0.05	0.4	−0.05	0.4	−0.39	<0.05
Waist circumference (cm)	−0.05	0.54	0.04	0.31	−0.60	<0.001
HDL-c (mg/L)	0.14	0.59	0.09	0.13	0.47	<0.001
TG (mg/dL)	−0.03	0.6	−0.02	0.68	−0.45	<0.001
Glucose (mg/dL)	0.06	0.29	−0.06	0.32	−0.41	<0.001
HbA1c (mmol/mol)	0.14	0.06	0.09	0.2	−0.36	<0.005
25-OH Vitamin D(ng/mL)	0.2	<0.001	0.15	<0.01	0.55	<0.001
CVR (Framingham)	−0.06	0.5	0.13	0.5	−0.61	<0.001
FLI	−0.11	0.8	−0.03	0.7	−0.62	<0.001
VAI	−0.05	0.4	−0.04	0.5	−0.45	<0.001
HSI	0.08	0.2	−0.05	0.4	−0.39	<0.001
NFS	0.02	0.7	−0.02	0.7	−0.56	<0.001

Data are presented as non-parametric Spearman correlation (r) and *p*-values. Abbreviations: Med Diet Score, MDS, Chrono Med Diet Score, CMDS; Body Mass Index, BMI; HDL-C; triglycerides, TG; glycosylated hemoglobin, HbA1c; cardiovascular risk, CVR; Fatty Liver Index, FLI; Visceral Adiposity Index, VAI; Hepatic Steatosis Index, HISNAFLD Score, NFS.

## Data Availability

Data presented in this study are available upon request from the corresponding author. The data are not publicly available due to ethical issues.

## Supplementary Materials

The following supporting information can be downloaded at: https://www.mdpi.com/article/10.3390/nu15081910/s1, Figure S1: The Mediterranean Diet Score questionnaire; Figure S2: The MEDI-LITE score questionnaire.
