# Supplementary material for: Identification of a Novel Score for Adherence to the Mediterranean Diet That Is Inversely Associated with Visceral Adiposity and Cardiovascular Risk: The Chrono Med Diet Score (CMDS)
Source: Nutrients. 2023 Apr 15;15(8):1910. doi: 10.3390/nu15081910 (PMC10141687; doi:10.3390/nu15081910)
Supplement: Supplementary file 1 [file nutrients-15-01910-s001.zip › nutrients-2308785-supplementary.pdf]

	TIMES PER WEEK					
	NEVER	1-4	5-8	9-12	13-18	>18
NON-REFINED CEREALS (whole grain bread, pasta, rice, etc.)	0	1	2	3	4	5
POTATOES	0	1	2	3	4	5
FRUITS	0	1	2	3	4	5
VEGETABLES	0	1	2	3	4	5
LEGUMES	0	1	2	3	4	5
FISH	0	1	2	3	4	5
RED MEAT AND MEAT PRODUCTS	5	4	3	2	1	0
POULTRY	5	4	3	2	1	0
FULL FAT DAIRY PRODUCTS (cheese, yoghurt and milk)	5	4	3	2	1	0
OLIVE OIL IN COOKING	0	1	2	3	4	5
ALCOHOLIC BEVERAGES (ml/day, 100ml = 12g ethanol)	5	4	3	2	1	0

Figure S1. The Mediterranean Diet Score questionnaire. (*Panagiotakos, D.B.; Pitsavos, C.; Arvaniti, F.; Stefanadis, C. Adherence to the Mediterranean food pattern predicts the preva-lence of hypertension, hypercholesterolemia, diabetes and obesity, among healthy adults; the accuracy of the MedDietScore. Prev Med 2007, 44, 335-340, doi:10.1016/j.ypmed.2006.12.009.*)

FRUIT<br>*1 portion: 150g*	<1 portion/d<br>**0**	1-2 portion/d<br>**1**	>2 portion/d<br>**2**
VEGETABLES<br>*1 portion: 100g*	<1 portion/d<br>**0**	1-2,5 portion/d<br>**1**	>2,5 portion/d<br>**2**
LEGUMES<br>*1 portion: 70g*	<1 portion/w<br>**0**	1-2 portion/w<br>**1**	>2 portion/w<br>**2**
CEREALS<br>*1 portion: 130 g*	<1 portion/d<br>**0**	1-1,5 portion/d<br>**1**	>1,5 portion/d<br>**2**
FISH<br>*1 portion:100g*	<1 portion/w<br>**0**	1-1,5 portion/w<br>**1**	>2 portion/w<br>**2**
MEAT AND MEAT PRODUCTS<br>*1 portion 80g*	<1 portion/d<br>**2**	1-1,5 portion/d<br>**1**	>1,5portion/d<br>**0**
DAIRY PRODUCTS<br>*1 portion: 180g*	<1 portion/d<br>**2**	1-1,5 portion/d<br>**1**	>1,5portion/d<br>**0**
OLIVE OIL	Occasional use<br>**0**	Frequent use<br>**1**	Regular use<br>**2**
ALCOHOL<br>*1 A.U= 1 glass of wine; 12g of alcohol*	<1 A.U./d<br>**1**	1-2 A.U./d<br>**2**	>2 A.U./d<br>**0**

Figure S2. The MEDI-LITE score questionnaire. (*Sofi, F.; Dinu, M.; Pagliai, G.; Marcucci, R.; Casini, A. Validation of a literature-based adherence score to Mediterranean diet: the MEDI-LITE score. Int J Food Sci Nutr 2017, 68, 757-762, doi:10.1080/09637486.2017.1287884.*)
